# How the partial-slip boundary condition can influence the interpretation of the DLS and NTA data

**DOI:** 10.1007/s10867-020-09546-5

**Published:** 2020-04-25

**Authors:** Vladimir P. Zhdanov

**Affiliations:** 1 Section of Biological Physics, Department of Physics, Chalmers University of Technology, Göteborg, Sweden; 2 Boreskov Institute of Catalysis, Russian Academy of Sciences, Novosibirsk, Russia

**Keywords:** Size of nanoparticles, Dynamic light scattering, Nanoparticle tracking analysis, Stokes-Einstein relation, Partial-slip boundary condition

## Abstract

Dynamic light scattering (DLS) and nanoparticle tracking analysis (NTA) are widely used to determine the size of biological nanoparticles in liquid. In both cases, one first measures the nanoparticle diffusion coefficient and then converts it to the nanoparticle radius via the Stokes-Einstein relation. This relation is based on the no-slip boundary condition. Now, there is evidence that this condition can be violated in biologically relevant cases (e.g., for vesicles) and that in such situations the partial-slip boundary condition is more suitable. I show (i) how the latter condition can be employed in the context of DLS and NTA and (ii) that the use of the former condition may result in underestimation of the nanoparticle radius by about 10 nm compared with the nominal one.

Biological nanoparticles, e.g., lipid vesicles, micelles, bicelles, virions, and lipid or colloidal nanoparticles employed for drug or RNA delivery, are usually suspended in liquid, and the determination of their size is an important step in their studies and/or the use in applications. Although in general the measurement of size can be done by employing various techniques (reviewed in [1]), practically, it is often performed by using DLS and/or NTA. DLS is based on the analysis of the correlations in the normalized intensity of the scattered light (reviewed in [2–4]). In the simplest case of monodisperse particles with single scattering, the corresponding correlation function is, for example, represented as:

1 $$ g^{}_{2}(q,\tau )=1+\exp(-2D q^{2} \tau), $$

where *D* is the particle diffusion coefficient, *q* is the amplitude of the scattering vector, and *τ* is the time interval between measurements. NTA is complementary to DLS, and its use is based on the conventional relation between the mean-square displacement of particles and time (reviewed in [5–8]):

2 $$ \langle {\Delta} x^{2} \rangle + \langle {\Delta} y^{2} \rangle = 4 D t, $$

or the corresponding Green function:

3 $$ G(r,t)=G({\Delta} x,t)G({\Delta} y,t), $$

where $G({\Delta } x,t)=(4\pi Dt)^{-1/2}\exp (-{\Delta } x^{2}/4Dt)$ (*G*(Δ*y*,*t*) is defined by analogy). Both these techniques measure *D*. The nanoparticle size or, more specifically, hydrodynamic radius (*R*) is then determined by using the Stokes-Einstein relation between *D* and *R*:

4 $$ D =\frac{k_{\mathrm{B}}T}{6\pi \eta R}, $$

where *k*_B_ is the Boltzmann constant, *T* is temperature, and *η* is the liquid viscosity. In the case of spherical nanoparticles (such particles are under consideration below), the hydrodynamic radius is considered to be equal to the nominal radius. Other ingredients and/or extensions of the theory underlying DLS and NTA are focused on the details of calculation of the correlation function (including, e.g., multiple scattering [3]), details of calculation of the mean-square displacement (including, e.g., specification of the lower and upper limits [8]), ways of determination of the nanoparticle-sized distribution (including the iterative histogram method [9, 10] and curve-fitting [11, 12]; see also Ref. [13]), and the factors (e.g., long-range electrostatic interaction [14]) complicating diffusion of nanoparticles. For the comparison of the DLS and NTA results obtained for monodisperse nanoparticles of independently characterized size, one can see, for example, Refs. [6, 13, 15].

Biological nanoparticles (e.g., vesicles, micelles, and enveloped and non-enveloped virions) are often fully or nearly spherical, and for DLS and NTA characterization of such particles the Stokes-Einstein relation (4) is accepted axiomatically (see the above-mentioned references). In fact, the use of this relation for the determination of the size of nanoparticles can be viewed as one of the basic principles of DLS and NTA. Its textbook derivation is based on the detailed balance principle relating the diffusion coefficient and mobility:

5 $$ D= k_{\mathrm{B}}T \alpha, $$

and the Stokes relation between the force and the particle-drift velocity:

6 $$ F = 6\pi \eta R v . $$

For the mobility, this relation yields

7 $$ \alpha =\frac{1}{6\pi \eta R} . $$

Substituting this expression into (5) results in (4).

The derivation of the Stokes relation (6) is known to be based on the no-slip condition at the interface between a nanoparticle and liquid. In fact, this condition implies two conditions concerning the normal and tangential velocities. Both of them should vanish. In physics, the no-slip boundary condition has long been considered to be obviously correct and is still often (e.g., in the literature related to DLS and NTA) accepted without discussion. During the past two decades, there were attempts to scrutinize experimentally whether this condition really holds (see, for example, the article by Joseph and Tabeling [17], published in 2005 and containing a discussion of earlier studies, and more recent articles [18–24]). The outcome of the available studies performed at macroscopic interfaces is that it can be violated, and in such cases the condition for the velocity along the interface should be reformulated in terms of a partial slip boundary as:

8 $$ \left . v (0) = b  \frac{\partial v}{\partial z}\right |_{z=0} , $$

where *z* is the normal coordinate (*z* = 0 corresponds to the flat interface), *v*(0) is the velocity at the interface, and *b* > 0 is the length corresponding to extrapolation of the velocity, *v*(*z*), to the region with *z* < 0 down to *v* = 0 (Fig. 1).

**Fig. 1:**
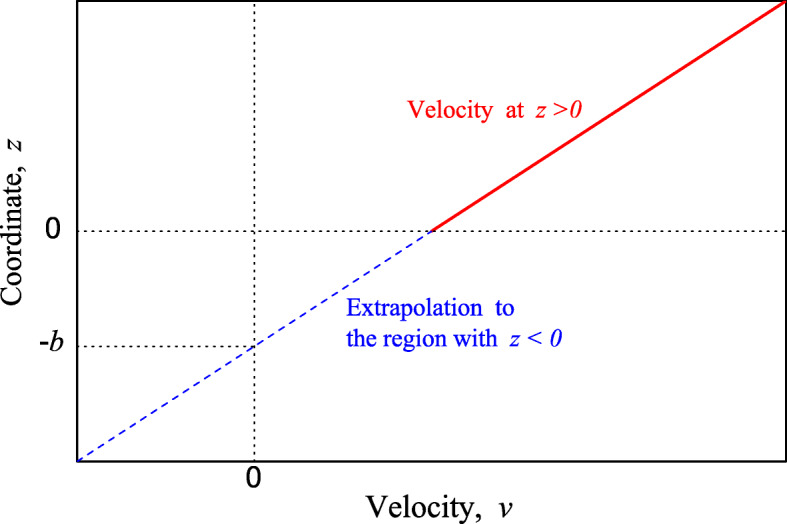
Velocity of liquid (solid line) along the liquid–solid interface as a function of the coordinate normal to the interface. The dashed line shows extrapolation of the velocity to the region below the interface so that *v* = 0 at *z* = −*b*. Note that in reality *b* is positive (or zero) and accordingly − *b* is negative

Formally, condition (8) means that the liquid velocity is finite at the interface. Physically, the properties of the liquid just near the interface are different compared with those far from the interface. The scale of the size of this region can be comparable with 1 nm, and the difference of the properties is expected to influence what happens there, i.e., in a thin layer of thickness *δ* ≤ 1 nm. From this perspective, condition (8) is expected to correspond to the region just above this layer, i.e., at $z \simeq \delta $ rather than literally at *z* = 0 (this aspect is discussed, e.g., in Ref. [16]), and one could expect that condition (8) would work provided *b* ≫ *δ*. If however *b* is small (comparable with *δ*), the velocity *v*(0) determined by (8) is nearly negligible, and condition (8) becomes equivalent to the conventional no-slip boundary condition as it is expected to be in this limit. Thus, condition (8) can be used down to *b* = 0. For the attempts to construct microscopic models allowing interpretation of *b*, one can read, for example, recent articles [25, 26] and references therein.

In the theoretical literature, one can find a generalization of the boundary condition (8) to spherically shaped particles (Sec. 4.20 in [27]). It implies a linear relation between the tangential velocity of liquid relative to the interface and the stress:

9 $$ \left.\beta v_{\theta} = {\Pi}_{r \theta} \right |_{r=R} , $$

where *r* and *𝜃* are the polar coordinates, and *β* is the coefficient of proportionality. In general, the stress is defined as

10 $$ {\Pi}_{r \theta}=\eta \left [ \frac{1}{r}\frac{\partial v_{r}}{\partial \theta} +r\frac{\partial}{\partial r} \left (\frac{v_{\theta}}{r} \right ) \right ]. $$

At the interface, the radial velocity *v*_*r*_ vanishes, and accordingly the first term in the right-hand part of (10) can be dropped, and accordingly (9) can be rewritten as:

11 $$ \left (\beta + \frac{\eta}{r}\right ) v_{\theta} = \eta  \frac{\partial v_{\theta}}{\partial r}. $$

If a nanoparticle is large so that

12 $$ \beta\gg \eta /r, $$

*η*/*r* can in (11) be neglected. In this limit, and condition (11) should be equivalent to (8). This means that *β*, *η*, and *b* are related as

13 $$ \eta /\beta = b . $$

With this relation, condition (12) can be rewritten as

14 $$ r \gg b. $$

The latter condition is often more convenient because *b* can be measured experimentally (see Table 1 below).

**Table 1:** Results of measurements of *b* for some materials (in historical order). The accuracy of such measurements can usually be debated

Material	*b* (nm)	Ref.
Glass	50 ± 50	[17]
Chlorodimethyloctylsilane	57 ± 100	[17]
Borosilicate glass	0	[18]
Octadecyltrichlorosilane	19 ± 2	[18]
DPPC lipid	$\simeq 10$	[19]
Silanized glass	45 ± 15	[20]
DOPC lipid	$\simeq 6$	[21]
Graphite	$\simeq 10$	[22]
Mica	0	[23]
Octadecyltrichlorosilane	$\simeq 28$	[23]

With partial-slip boundary condition (11), the steady-state motion of liquid around a nanoparticle can be described analytically (Sec. 4.20 in [27]), and the force acting on a nanoparticle is given by

15 $$ F = 6\pi \eta R v \frac{R+2b}{R+3b}. $$

The corresponding expressions for the mobility and diffusion coefficient (according to (5)) are as follows:

16 $$ \alpha = \frac{1}{6\pi \eta R} \frac{R+3b}{R+2b}, $$

17 $$ D = \frac{k_{\mathrm{B}}T}{6\pi \eta R} \frac{R+3b}{R+2b}. $$

For *b* ≪ *R* and *b* ≫ *R*, we respectively have

$$ \frac{R+2b}{R+3b}\simeq 1 - \frac{b}{R}  \text{and} \frac{R+2b}{R+3b}\simeq \frac{2}{3}. $$

In these limits, expression (17) is reduced, respectively, to:

18 $$ D \simeq \frac{k_{\mathrm{B}}T}{6\pi \eta (R-b)} , $$

19 $$ D \simeq \frac{k_{\mathrm{B}}T}{4\pi \eta R} . $$

In DLS and NTA, as already noted, the nanoparticle radius is determined by using relation (4) based on the no-slip boundary condition. If in reality this condition is not valid, the use of relation (4) will result in a systematic error in the determination of the nanoparticle radius. In particular, these techniques will yield the apparent nanoparticle radius, *R*_ap_. According to (17), (18), and (19), this radius is expected to be related with the nominal radius, *R*, as:

20 $$ R_{\text{ap}} = R \frac{R+2b}{R+3b},  \text{or} $$

21 $$ R_{\text{ap}} \simeq R-b  \text{provided}  b \ll R,  \text{and} $$

22 $$ R_{\text{ap}} \simeq 2R/3  \text{provided}  b \gg R. $$

In the context of experiments, these relations between the apparent and nominal nanoparticle radii are valid provided there are no other systematic errors in measurements. What they indicate is that the apparent radius may be smaller that the nominal radius (Fig. 2). This is the main message of this communication.

**Fig. 2:**
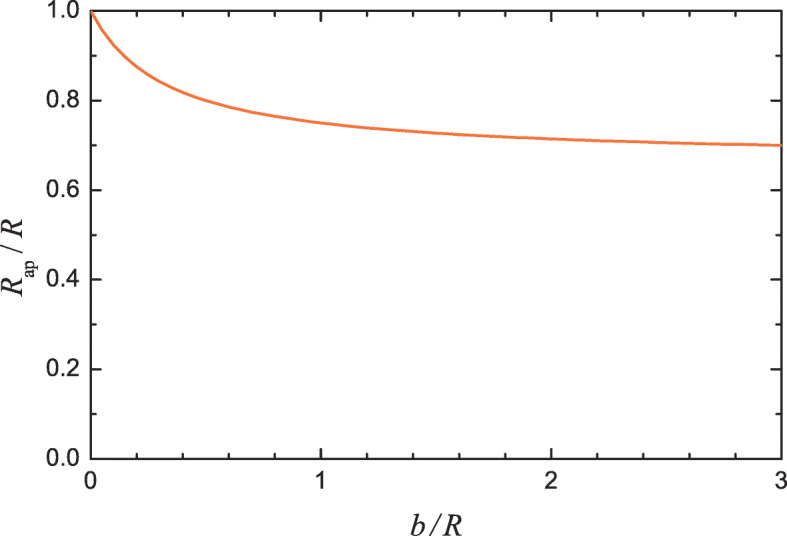
Apparent nanoparticle radius as a function of the ratio of *b* and the nominal radius (according to (20)). The results shown in this figure imply that *b* is independent of *R*. In principle, however, *b* can depend on *R*

The extent of reduction of the apparent radius compared with the nominal radius depends on *b*. Physically, *b* is expected to be appreciable for hydrophobic materials. Accurate measurements of *b* are still challenging. Some results of such measurements performed at macroscopic surfaces are collected in Table 1. One can see that *b* may be appreciable for biologically relevant materials. Lipid molecules, for example, are composed of a hydrophilic head and two hydrophobic hydrocarbon chains so that the exterior part of lipid bilayers is hydrophilic [28], and accordingly one might expect that for them *b* would be negligible. The corresponding experiments indicate, however, that *b* is about 10 nm [19, 21]. The accuracy of measurements of *b* is unfortunately not high. With this reservation, the ratio *R*_ap_/*R* can be calculated with this value of *b* as a function of *R* for the values of *R* typical for small vesicles (Fig. 3). Roughly, this value of *b* characterizes the scale of how the no-slip boundary condition can influence the DLS- and NTA-measured size of vesicles, and accordingly it has been indicated in the abstract.

**Fig. 3:**
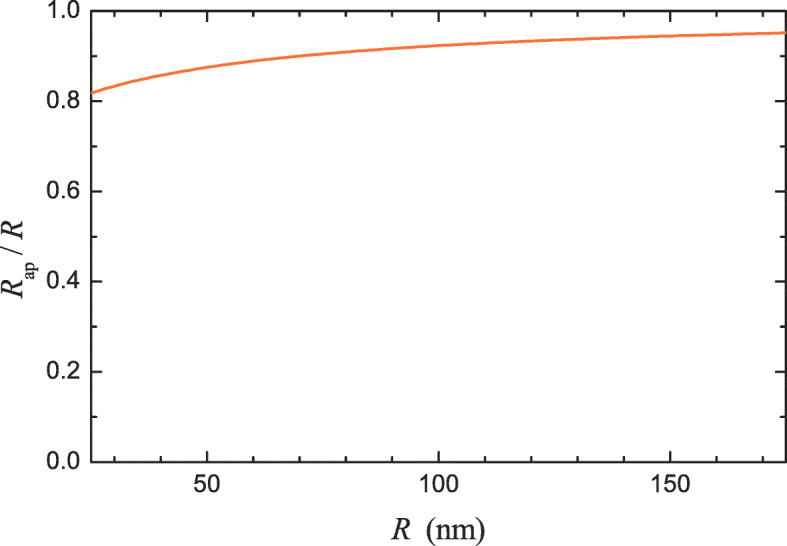
Apparent nanoparticle radius as a function of the nominal radius for *b* = 10 nm (according to (20))

Another interesting aspect is whether the difference between the apparent and nominal nanoparticle radii is observed in the experiments aimed at validation of DLS and/or NTA. Looking through the corresponding studies, one can conclude that from this perspective the available results are somewhat contradictory. In a few studies, for example, monodisperse polystyrene nanobeads of specified size were used to prove the accuracy of DLS and/or NTA [6, 13, 15]. For sizes from 60 to 1000 nm, DLS is found to overestimate slightly (by about 10%) the size whereas NTA reproduces the nominal size (Table 1 in [6]). The results obtained for 100-nm-sized beads [13] are similar. For sizes of 92, 269, and 343 nm, DLS is reported to overestimate slightly (by about 10%) the size while NTA underestimates the size (by up to about 30%) (Fig. 1 in [15]). Although, as already noted, the results of these experiments and some other similar experiments reported by different groups are not in full mutual agreement, the scale of the difference between the measured and nominal sizes is comparable with that predicted by my analysis focused on the partial-slip boundary condition. This is an additional argument indicating that the related corrections can be important along with some other corrections which should be discussed in each specific case. The latter is beyond my goals because such discussions are expected to include tiny details of measurements (which are often not published with the data (see, for example, the discussion in [29])) and accordingly should be done by the corresponding experimentalists.

Finally, one can notice that what happens near the interface between a nanoparticle and liquid or, more specifically, the value of *b* in condition (8) may obviously depend on the presence of surfactant there [30, 31]. It can influence the results of measurements undesirably because the corresponding surface coverage can be appreciable even in the presence of traces of these species in solution. In the other way around, surfactants can be used to modify intentionally the interface in order to reach one goal or another.

Taken together, the results presented and discussed in this communication extend the basis for the interpretation of DNS and NTA measurements. The key conclusion is that the partial-slip boundary conditions can be relevant in the context of DNS and NTA measurements of size of biological nanoparticles, e.g., vesicles. It does not exclude, however, that the interpretation of such measurements can be complicated by other factors.
